# Safety of individualized herbal medicine for dysmenorrhea: pharmacovigilance from South Korea’s national pilot

**DOI:** 10.3389/fphar.2026.1722249

**Published:** 2026-02-09

**Authors:** Minjung Park, Yongjoo Kim, Nan-He Yoon, Kyeore Bae, Junhyeok Yi, Sujin Kim

**Affiliations:** 1 Department of Preventive Medicine, College of Korean Medicine, Gachon University, Seungnam, Republic of Korea; 2 Department of Korean Medical Science, College of Korean Medicine, Sangji University, Wonju, Republic of Korea; 3 Division of Social Welfare and Health Administration, Wonkwang University, Iksan, Republic of Korea; 4 National Agency for Development of Innovative Technologies in Korean Medicine, National Institute for Korean Medicine Development, Seoul, Republic of Korea; 5 Korea Institute for Health and Social Affairs, Sejong, Republic of Korea

**Keywords:** claims data, coarsened exact matching, difference-in-differences, drug safety, dysmenorrhea, herbal medicine

## Abstract

**Background:**

In 2020, South Korea’s National Health Insurance (NHI) began reimbursing individually prescribed herbal medicine decoctions (HMDs) for dysmenorrhea, highlighting the need for robust post-coverage safety monitoring using national claims data.

**Objective:**

To evaluate the safety of reimbursed HMDs for dysmenorrhea and demonstrate a claims-based pharmacovigilance approach to inform benefit management and clinical guideline integration.

**Methods:**

A retrospective cohort study was conducted using the Health Insurance Review & Assessment (HIRA) national claims database from 2015 to 2022. HMD users were coarsened-exact-matched to nonusers on sociodemographic and clinical characteristics. Difference-in-differences models compared pre- and post-coverage outcomes at 1, 3, 6, and 11 months. Primary outcomes were hepatotoxicity and renal failure; secondary outcomes included allergic reactions, emergency department visits and hospitalizations.

**Results:**

The matched cohort comprised 8,989 HMD users and 8,989 nonusers. Reimbursed HMDs were not associated with increased risk of severe adverse events at any time point. At 3 months, DID estimates were −2.6 (95% CI −411.8, 406.6) for hepatotoxicity and −7.2 (95% CI −402.3, 388.0) for renal failure, with consistent safety patterns across endpoints and sensitivity analyses.

**Conclusion:**

Coverage of individually prescribed HMDs under South Korea’s NHI system was not associated with elevated serious safety risks for dysmenorrhea. These findings support continued reimbursement and provide a scalable template for claims-based pharmacovigilance using standardized endpoints, routine signal detection, and feedback to benefit design and clinical practice.

## Background

Menstrual pain is a common gynecological condition experienced by 50% of menstruating individuals, significantly affecting their quality of life and contributing to socioeconomic losses (Gebeyehu et al., 2017). Untreated dysmenorrhea can interfere with daily activities, education, and work productivity (Proctor and Farquhar, 2006; Dixon et al., 2024; Mizuta et al., 2023). Severe dysmenorrhea may indicate underlying abnormalities, including endometriosis or uterine fibroids; delayed diagnosis can potentially affect future reproductive health (Park, 2022). Among adolescents, dysmenorrhea is a leading cause of school absenteeism; in adulthood, it results in a considerable reduction in labor productivity, leading to considerable socioeconomic losses (Slab, 2003).

Clinically it is defined as pain experienced in the lower abdomen and suprapubic region during or immediately before or after menstruation. It is categorized into primary dysmenorrhea (PD), which occurs without a specific organic pelvic lesion (N94.4), and secondary dysmenorrhea, caused by distinct pelvic pathology. The primary etiology of PD involves intense uterine contractions that generate high intrauterine pressure and restrict blood flow, resulting in ischemic pain. This mechanism is substantiated by high concentrations of prostaglandin (PG) F2α and PG E2 found in the endometrium and menstrual fluid, which contribute to uterine contraction, along with vasopressin, another factor known to cause uterine and vascular constriction (Society of Korean Medicine Gynecology and Lee, 2021).

Conventional treatments for dysmenorrhea include nonsteroidal anti-inflammatory drugs and hormonal contraceptives. However, nonsteroidal anti-inflammatory drugs are ineffective in 20%–25% of patients; their long-term use can lead to liver, kidney, and digestive disorders (Navvabi et al., 2012). Hormonal contraceptives are not suitable for patients planning pregnancy and can cause nausea, vomiting, edema, and an increased risk of venous thromboembolism, making their long-term use challenging (Wong et al., 2009).

In Korean Medicine, herbal treatments are widely used to manage dysmenorrhea, with individually prescribed decoction-type formulations being the most common approach. Since 2016, the Korean government has supported the development of evidence-based clinical practice guidelines (CPGs) for Korean medicine and promoted standardized, pattern-based prescribing (Shin et al., 2023). Per the Dysmenorrhea CPG, the primary approach is individually prescribed decoction-type herbal medicine tailored to specific patterns of disharmony, including Qi Stagnation and Blood Stasis, Cold-Damp Congealing, Qi and Blood Deficiency, and Liver–Kidney Deficiency (Society of Korean Medicine Gynecology and Lee, 2021).

Several studies have highlighted the potential benefits of traditional herbal medicines in managing dysmenorrhea. A Cochrane review encompassing 39 randomized controlled trials involving 3,475 patients found that Chinese herbal medicine significantly improved pain relief and overall symptoms compared with placebo, no treatment, or conventional therapies (Zhu et al., 2008). According to the Dysmenorrhea CPG, herbal medicine demonstrates a relatively excellent therapeutic effect in treating primary dysmenorrhea for pain reduction in comparison to NSAIDs, and reports a lower or similar incidence of adverse events compared to conventional Western medicines (NSAIDs or oral contraceptives) or placebo (Society of Korean Medicine Gynecology and Lee, 2021). Additionally, several studies have also provided robust evidence supporting the effectiveness of herbal medicines (Li et al., 2020; Lee et al., 2016a; Daily et al., 2015; Lee et al., 2016b; Jang et al., 2009).

Traditional medicines are used in most health systems worldwide: 170 of the World Health Organization (WHO)’s 194 Member States report traditional medicine (TM) use and have asked for robust evidence to guide policy, practice, and regulation (World Health Organization, 2025). WHO’s Traditional Medicine Strategy emphasizes integrating TM within health services and universal health coverage (UHC) while ensuring safety and quality through evidence-informed policies (Burki, 2025; Hoenders et al., 2024).

Recognizing its clinical and policy importance, the Korean government has included dysmenorrhea in a national pilot initiative expanding National Health Insurance (NHI) coverage for herbal medicines since 2020. The benefit package covered 50% of prescription costs and provided an herbal medicine decoction (HMD) to treat and manage chronic conditions, including dysmenorrhea. Eligible patients could receive HMD treatment for up to 10 days per year (Ministry of Health and Welfare, 2020), which is expected to improve accessibility and quality of life in the future (Choi and Kim, 2023).

In implementing the national pilot program, timely assessment of both effectiveness and safety is decisive for program success and scale-up. Although Kwon et al. (2024) reviewed clinical studies of herbal medicines commonly prescribed by Korean Medicine Doctors (KMD) and reported reassuring hepatic and renal profiles, such trial-based evidence may not reflect routine care. Decoction-type prescriptions are fully individualized, vary by formulation and dose, and are delivered to patients with heterogeneous ages, comorbidities, and concomitant medication use—conditions that heighten the potential for rare or context-specific adverse events. Accordingly, immediate, claims-enabled pharmacovigilance at program launch—not after expansion—is essential: standardized coded safety endpoints, routine signal detection at short intervals (e.g., monthly), and pre-specified escalation to targeted chart review can verify safety in real time and inform rapid adjustments to benefit criteria and clinical guidance. This approach ensures that coverage decisions remain evidence-responsive while safeguarding patients in real-world practice.

Therefore, we conducted a difference-in-differences (DID) natural experimental study using the Korean National Health Insurance Review and Assessment (HIRA) claims data, comparing changes before and after the intervention across individuals who shared similar medical and demographic characteristics, except for participation in the HMD benefit expansion initiative. This approach aligns with the increasing body of research in the realm of drug safety investigations using real-world data, including claims data, which includes an extended range of clinical safety outcome measures (Corrigan-Curay et al., 2018; Lavertu et al., 2021). The aim of this study was to determine the safety of HMD for dysmenorrhea by comparing patients with dysmenorrhea who underwent HMD treatment through the benefit expansion initiative with those who did not, utilizing real-world data from the national Korean HIRA Database.

## Methods

### Data source

This study analyzed claims data from the HIRA service in South Korea, covering the period from January 1, 2015, to February 28, 2022. We used data from 2015 onward to capture patients’ medical histories. Earlier data were used only for the look-back period and were not included in the DID analysis. South Korea’s NHI system operates as a universal public health insurance program that ensures coverage for nearly all citizens. HIRA oversees claims reviews and quality assessments for medical services under the NHI, compiling healthcare utilization data for the country’s population of approximately 50 million. The HIRA database contains patient demographics and inpatient and outpatient medical records, including clinical diagnoses coded under the International Classification of Diseases, Tenth Revision (ICD-10), medical procedures, and prescription drug information (HIRA, 2023).

### Study population

The dysmenorrhea cohort included patients whose primary medical records were classified under codes N94.4 (Primary dysmenorrhea), N94.5 (Secondary dysmenorrhea), and N94.6 (Dysmenorrhea, unspecified) between November 1, 2020, and February 28, 2022. Participants were categorized as HMD users if they had received at least one HMD prescription through the initiative, whereas nonusers were defined as individuals who received care from a conventional Western medicine physician but did not participate in the HMD initiative. The dysmenorrhea cohort was defined based on the eligibility criteria of the HMD benefit-expansion pilot program. Concomitant use of Western medications or non-pharmacological therapies was permitted. The index date for HMD users was defined as the date of their first HMD prescription under the initiative, whereas for nonusers, a single dysmenorrhea-related outpatient visits after cohort entry, which began on November 1, 2020, was randomly selected as the index date. Random selection was performed using a uniform random number assignment, such that each qualifying visit had an equal probability of being selected as the index date. To maintain cohort integrity, HMD users who had visited a KMD for conditions other than dysmenorrhea were excluded, as were nonusers who had consulted a KMD for any reason within 1 year following the index date. Patients were subsequently followed up for a period of 1 year from their respective index dates. The nationwide single-payer claims system ensured complete capture of all reimbursed healthcare encounters, resulting in no missing data for study variables. The study design and reporting followed the STROBE (Strengthening the Reporting of Observational Studies in Epidemiology) guidelines. The study design was informed by our previous work (Kim et al., 2025).

### Exposure assessment

The duration-response relation was evaluated by calculating the cumulative exposure duration over a 30-day period from the index date. Some patients received their second prescription immediately after completing the first whereas others did not; therefore, we used a 30-day window to capture cumulative exposure in a standardized manner. This was measured using the proportion of days covered (PDC), which was determined by dividing the total number of prescription days within the 30-day window by the total number of days during that period. Under the NHI system, the first visit allows for a 10-day prescription with a 50% co-payment. Patients may receive an additional prescription at full cost, as the NHI does not subsidize a second prescription. KMDs are required to report second prescriptions for dysmenorrhea even if they are paid full by patients (Ministry of Health and Welfare, 2020). Prescription records were included in the HIRA database. Given that the initiative permits up to two HMD prescriptions per patient, HMD users were classified into one-time users (PDC <0.34) and two-time users (PDC ≥0.34), reflecting receipt of a single versus the maximum number of treatment episodes. This categorization was intended to align with both the clinical pattern of dysmenorrhea management and the structural constraints of the pilot program, rather than to represent continuous long-term use.

### Outcome definition

The primary outcomes included hepatotoxicity and renal failure and were identified through inpatient or outpatient diagnoses based on ICD-10 codes. While hepatotoxicity was defined using ICD-10 code K71 based on previous literature (Kim and Jang, 2022), renal failure was defined using ICD-10 codes N10, N12, N14.1, N14.2, N15.8, N15.9, and N17.0-N17.9, selected in consultation with an internal medicine specialist in Korean medicine due to the lack of established references in prior research.

The secondary outcomes included allergic drug reactions, hospital admissions (excluding Korean medicine hospitals), and emergency department visits. Allergic drug reactions were defined by inpatient or outpatient diagnoses using ICD-10 codes (D72.1, L27.0, L27.1, L29.8, L29.9, L50.0, L50.9, R21, T78.2, T78.3, T78.4, T88.6, and T88.7) following the criteria established in studies (Saff et al., 2019; Shin et al., 2020).

**FIGURE 1:**
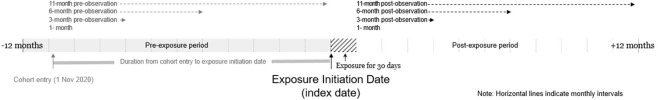
Study design and timing of pre- and post-intervention periods for difference-in-differences analysis.

Outcome assessments were conducted at 1st, 3rd, 6th, and 11th months from the index date, with measurements beginning 30 days after the index date. Pre-intervention outcomes were evaluated at the same time intervals, starting 30 days after the 1-year mark preceding the index date (Figure 1).

### Potential confounders: matching variables

To reduce potential bias owing to confounders, this study matched the treatment group with a control group with similar sociodemographic characteristics and health conditions. The sociodemographic characteristics included age and residence. The duration of dysmenorrhea at the time of the index date was measured in years, ranging from <1 year to >5 years.

The Charlson Comorbidity Index was used to assess health conditions. The Charlson Comorbidity Index is a weighted index ranging from one to six and is calculated based on the presence of 17 comorbidities identified using ICD-10 codes in the 1 year preceding the index date. A score of zero represents no comorbid conditions, whereas higher scores indicate an increased risk of comorbidities. The additional matching variables included myocardial infarction, congestive heart failure, peripheral vascular disease, cerebrovascular disease, dementia, liver disease, diabetes, hemiplegia or paraplegia, renal disease, and cancer. The use of acetaminophen or ibuprofen was defined by the duration of prescription over the 1 year before the index date.

Furthermore, this study used information on allergic responses, hepatic failure, and renal failure based on the corresponding ICD-10 codes from the claims data, as well as information on hospitalization, emergency service utilization, and Korean medicine healthcare service use during the 1 year preceding the index date for each participant. In addition, we included emergency visits during the 6-month period beginning 1 year before the index date to strengthen the plausibility of the parallel-trends assumption in the DID analysis, as the two groups exhibited differing pre-intervention trends in emergency visits.

### Empirical strategy

We applied the coarsened exact matching (CEM) method along with a quasi-experimental DID design to compare the outcome incidence between HMD users and nonusers before and after the index date. CEM is a multidimensional matching algorithm that operates by segmenting continuous variables into discrete intervals or consolidating categorical variables into broader coarsened categories. This approach generates strata with identical coarsened values for the matching variables, ensuring that the matched data remain within common empirical support. Thus, CEM enhances the balance in the empirical distribution of the matching variables between the treatment and control groups. A key advantage of CEM is its ability to reduce dependence on model assumptions, thereby improving the robustness of causal inferences (Iacus et al., 2017; Kim et al., 2025).

We utilized CEM to account for potential biases arising from the exaggerated person-time of the comparator group and other confounders, focusing on the period between cohort entry and the index date. Each HMD user was matched with a nonuser with similar sociodemographic and medical characteristics.

Following the matching process, we applied the DID approach to estimate the effect of HMD use on the outcome incidence. This was achieved by comparing the change in outcome incidence among HMD users while offsetting any concurrent changes observed in nonusers. The DID estimator is formulated as follows:

$$In\;\left(\mathrm{Pr}\left(Y_{i}=1|X\right)/\left(1-\mathrm{Pr}\left(Y_{i}=1|X\right)\right)\right)=\beta_{0}+\beta_{1}{Post}_{i}+\beta_{2}{One\;prescription}_{i}+\beta_{3}{Two\;prescriptions}_{i}+\beta_{4}{One\;prescription}_{i}\cdot {Post}_{i}+\beta_{5}{Two\;prescriptions}_{i}\cdot {Post}_{i}+e_{i}$$

$Y_{i}$ is a binary outcome variable, including hepatotoxicity, renal failure, allergic drug reactions, hospital admissions, and emergency visits, taking a value of one if the individual experienced each event and zero otherwise. “One prescription” and “two prescriptions” are variables that take a value of one if an individual used HMD once and twice under the initiative, respectively, and zero otherwise. “Post” is a variable that takes a value of one if the outcome measurement was made in the post-policy period (after the index date) and zero otherwise. The interaction term coefficients, $\beta_{4}$ and $\beta_{5}$ , represent the estimates of interest, which indicate the DID effect on the outcome variable attributable to one-time and two-time prescriptions of HMD, respectively. Given the nature of the outcome variables, we used logistic regression and ran separate models for each outcome measure.

The DID analyses were valid only when the outcomes between the treatment and control groups exhibited parallel trends (Angrist and Pischke, 2009). We used interaction terms between treatment status and the period before the index date to assess whether each outcome indicator in HMD users had significantly different trends from those in nonusers, focusing specifically on the periods 1 and 2 years before (Supplementary Table S1). Data analyses were performed using SAS Enterprise Guide 7.1 (SAS Institute, Cary, NC, United States).

### Ethics

As this study utilized de-identified data provided by HIRA, which had been anonymized in accordance with strict confidentiality guidelines, it was exempt from ethical review by the Institutional Review Board (IRB number: SJIRB–Human–22–003).

## Results

**FIGURE 2:**
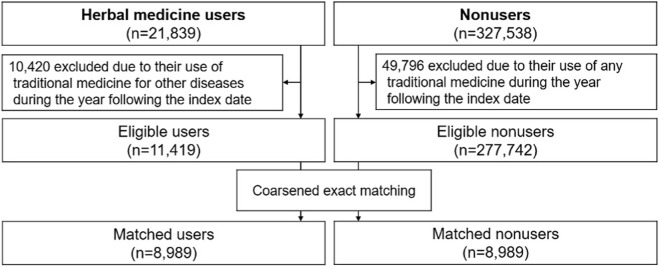
Selection of study participants and construction of matched cohort.

After conducting the CEM, the user and nonuser cohorts consisted of 8,989 patients each (Figure 2). The baseline characteristics of the users and nonusers in the CEM-matched cohorts, which were well balanced between the two groups, are presented in Table 1.

**TABLE 1:** Baseline characteristics of herbal medicine users and nonusers in matched patients with dysmenorrhea.

Characteristics^a^	Control (n = 8,989)	Treatment (n = 8,989)	*P value* ^b^
One-time prescription, no. (%)	-	7,870 (87.55)	NA
Two-time prescriptions, no. (%)	-	1,119 (12.45)	
Age, mean (SD), years	27.45 (10.21)	27.44 (10.29)	1.0
Region: Seoul	2472 (27.50)	2364 (26.30)	​
Busan	390 (4.34)	390 (4.34)	​
Incheon	471 (5.24)	471 (5.24)	​
Daegu	485 (5.40)	485 (5.40)	​
Gwangju	221 (2.46)	221 (2.46)	​
Daejeon	307 (3.42)	307 (3.42)	​
Ulsan	88 (0.98)	88 (0.98)	​
Gyeonggi	2790 (31.04)	2790 (31.04)	​
Gangwon	135 (1.50)	135 (1.50)	​
Chung-buk	180 (2.00)	180 (2.00)	​
Chung-nam	283 (3.15)	391 (4.35)	​
Heon-buk	229 (2.55)	229 (2.55)	​
Jeon-nam	110 (1.22)	110 (1.22)	​
Gyeong-buk	231 (2.57)	231 (2.57)	​
Gyeong-nam	495 (5.51)	495 (5.51)	​
Jeju	47 (0.52)	47 (0.52)	​
Sejong	55 (0.61)	55 (0.61)	​
Duration of dysmenorrhea, mean (SD), years	1.36 (1.03)	1.36 (1.03)	1.0
Duration from cohort entry, mean (SD), days	205.21 (143.55)	201.87 (143.85)	0.1
Allergic responses, no. (%)	203 (2.26)	203 (2.26)	1.0
Hepatic failure, no. (%)	0	0	NA
Renal failure, no. (%)	1 (0.01)	1 (0.01)	1.0
Admissions to hospitals, no. (%)	497 (5.53)	496 (5.52)	1.0
Emergency visits, no. (%)	153 (1.70)	154 (1.71)	1.0
Use of Korean medicine, no. (%)	2674 (29.75)	2674 (29.75)	1.0
Prescription duration of acetaminophen or ibuprofen, mean (SD), days	5.08 (8.86)	4.93 (12.74)	0.3
Charlson comorbidity index, mean (SD)	0.03 (0.19)	0.03 (0.19)	1.0
Myocardial infarction, no. (%)	0	0	NA
Congestive heart failure, no. (%)	0	0	NA
Peripheral vascular disease, no. (%)	1 (0.01)	1 (0.01)	1.0
Cerebrovascular disease, no. (%)	1 (0.01)	1 (0.01)	1.0
Dementia, no. (%)	0	0	NA
Liver disease, no. (%)	28 (0.31)	28 (0.31)	1.0
Diabetes, no. (%)	5 (0.06)	5 (0.06)	1.0
Hemiplegia or paraplegia, no. (%)	0	0	NA
Renal disease, no. (%)	0	0	NA
Cancer, no. (%)	13 (0.14)	13 (0.14)	1.0

^a^ Sex, age, duration of dysmenorrhea, and duration from cohort entry were measured at the time of the index date; the Charlson Comorbidity Index, presence of comorbidities, hepatic and renal failure, allergic responses, hospital admissions, emergency visits, and use of traditional medicine were identified in the year preceding the index date.

^b^ *P* values represent the significance of the chi-square test for sex and other variables with t-statistics.

Table 2 presents the coefficients for safety events associated with single and double prescriptions of HMD compared with those of nonuse in the CEM-matched cohort. HMD use did not increase the risk of hepatotoxicity or renal failure during the 1-, 3-, 6-, and 11-month observation periods, as the coefficients were insignificant for both one-time and two-time prescriptions. For instance, the DID effect estimates and 95% CIs comparing two-time users with nonusers were −2.6 (95% CI: −411.8, 406.6) for hepatotoxicity and −7.2 (95% CI: −402.3, 388.0) for renal failure. Moreover, the findings showed that HMD users with single or double prescriptions had risks of allergic drug reactions, admissions to Western medicine hospitals, and emergency visits similar to those of non-HMD users across all observation periods.

**TABLE 2:** Difference-in-differences analysis: safety events in herbal medicine users compared with nonusers during the 1-, 3-, 6-, and 11-month observation periods^a^.

Variables	One month	Three months	Six months	Eleven months
	***β*** coefficient (95% CI)	***β*** coefficient (95% CI)	***β*** coefficient (95% CI)	***β*** coefficient (95% CI)
Hepatic failure				
No. of events	1	2	4	4
One-time prescription	0.3 (−175.7 to 176.4)	0.3 (−204.9 to 205.6)	0.1 (−208.4 to 208.5)	0.1 (−208.4 to 208.5)
Two-time prescriptions	0.1 (−284.5 to 284.7)	0.1 (−331.7 to 331.9)	0.0 (−335.0 to 334.9)	0.0 (−335.0 to 334.9)
Post-period	3.0 (−194.0 to 200.0)	3.3 (−226.3 to 233.0)	6.4 (−228.9 to 241.7)	6.4 (−228.9 to 241.7)
One-time · post-period	5.5 (−206.9 to 217.9)	6.2 (−241.4 to 253.8)	3.7 (−252.6 to 260.0)	3.7 (−252.6 to 260.0)
Two-time · post-period	−2.3 (−353.3 to 348.7)	−2.6 (−411.8 to 406.6)	−6.2 (−454.7 to 442.2)	−6.2 (−454.7 to 442.2)
Renal failure				
No. of events	6	16	31	51
One-time prescription	0.0 (−177.1 to 177.1)	0.0 (−178.8 to 178.8)	6.1 (−166.6 to 178.7)	2.9 (−124.0 to 129.9)
Two-time prescriptions	0.0 (−281.1 to 281.1)	0.0 (−283.9 to 283.9)	−3.0 (−333.7 to 327.6)	−5.7 (−259.6 to 248.2)
Post-period	6.5 (−198.4 to 211.4)	7.2 (−199.7 to 214.1)	8.4 (−164.2 to 181.1)	6.0 (−121.0 to 132.9)
One-time · post-period	3.0 (−220.4 to 226.3)	3.5 (−222.0 to 229.1)	−5.9 (−178.5 to 166.8)	−2.8 (−129.7 to 124.2)
Two-time · post-period	−6.5 (−397.8 to 384.9)	−7.2 (−402.3 to 388.0)	2.6 (−328.0 to 333.2)	5.7 (−248.2 to 259.6)
Allergic responses				
No. of events	118	324	586	951
One-time prescription	0.4 (−0.3 to 1.1)	0.2 (−0.2 to 0.7)	0.0 (−0.3 to 0.2)	0.0 (−0.2 to 0.2)
Two-time prescriptions	−0.7 (−2.0 to 0.6)	−0.6 (−1.3 to 0.2)	−0.1 (−0.5 to 0.3)	0.0 (−0.3 to 0.3)
Post-period	0.6 (−0.3 to 1.4)	0.7 (0.3 to 1.2)	0.6 (0.3 to 0.9)	0.3 (0.1 to 0.5)
One-time · post-period	−0.4 (−1.3 to 0.5)	−0.3 (−0.8 to 0.2)	−0.1 (−0.4 to 0.3)	0.0 (−0.2 to 0.3)
Two-time · post-period	0.1 (−1.5 to 1.7)	0.2 (−0.7 to 1.1)	0.0 (−0.5 to 0.5)	−0.2 (−0.6 to 0.2)
Admissions to non-traditional medicine hospitals				
No. of events	260	678	1197	2020
One-time prescription	0.4 (−0.3 to 1.1)	−0.1 (−0.3 to 0.2)	−0.1 (−0.2 to 0.1)	0.0 (−0.2 to 0.1)
Two-time prescriptions	−1.2 (−2.5 to 0.1)	−0.1 (−0.5 to 0.2)	−0.1 (−0.3 to 0.2)	0.1 (−0.1 to 0.2)
Post-period	1.1 (0.4 to 1.8)	0.5 (0.3 to 0.8)	0.4 (0.2 to 0.6)	0.1 (0.0 to 0.2)
One-time · post-period	−0.5 (−1.3 to 0.3)	0.0 (−0.3 to 0.3)	0.0 (−0.2 to 0.2)	0.0 (−0.2 to 0.1)
Two-time · post-period	1.1 (−0.3 to 2.5)	0.0 (−0.5 to 0.5)	0.0 (−0.3 to 0.3)	−0.1 (−0.4 to 0.1)
Emergency visits				
No. of events	40	125	242	352
One-time prescription	3.6 (−139.7 to 146.9)	0.0 (−0.3 to 0.4)	0.1 (−0.2 to 0.3)	0.1 (−0.1 to 0.3)
Two-time prescriptions	−7.6 (−294.3 to 279.0)	−0.1 (−0.7 to 0.5)	−0.2 (−0.6 to 0.3)	−0.2 (−0.5 to 0.2)
Post-period	3.0 (−140.4 to 146.3)	−0.8 (−1.3 to −0.3)	−1.1 (−1.5 to −0.7)	−1.4 (−1.8 to −1.0)
One-time · post-period	−4.0 (−147.3 to 139.3)	−0.5 (−1.1 to 0.2)	−0.3 (−0.9 to 0.2)	−0.4 (−0.9 to 0.1)
Two-time · post-period	8.5 (−278.1 to 295.2)	0.6 (−0.4 to 1.5)	0.3 (−0.5 to 1.1)	0.1 (−0.6 to 0.9)

^a^ All analyses were conducted using 8,989 users and 8,989 matched nonusers, observed in both the pre-intervention and post-intervention periods (total N = 35,956 observations). No additional covariates were incorporated as all potential confounders exhibited very similar characteristics, as shown in Table 1.

Supplementary Table S1 shows the results of the robustness check assessing the parallel trends assumption for the DID analysis. The estimated pre-intervention trends for both one-time and two-time HMD users did not differ significantly from those of nonusers across all outcome measures, supporting the validity of the parallel trend assumption.

## Discussion

This study employed a cohort design utilizing real-world data and applied CEM with a DID natural experimental analysis to assess the safety of HMD for dysmenorrhea. Drawing on data from the Korean HIRA database, our findings suggest that individuals who received HMD treatment under the benefit-expansion initiative exhibited no significant increase in risk was detected, including healthcare utilization related to hepatic and renal toxicity, allergic reactions, emergency department visits, and hospitalizations, compared with matched individuals who did not participate in the initiative, across the 1-, 3-, and 6-month follow-up periods.

In Korean medicine, herbal treatments are tailored to individual health conditions based on traditional medical principles. Herbal decoctions are the preferred formulation because they maximize the benefits of personalized prescriptions, distinguishing them from standardized herbal extracts. Accordingly, the South Korean government launched national pilot initiatives to provide insurance coverage for decoction-type herbal prescriptions. This initiative not only underscores the individualized approach inherent in traditional Korean medicine but also highlights the government’s proactive efforts to integrate traditional therapies into the national healthcare system. However, as these treatments become more widely used, continuous safety monitoring remains crucial.

Lin et al. (2022) reported, based on a retrospective review of 338 women with ultrasonography- or surgery-confirmed endometriosis-related menstrual pain, that herbal medicine was associated only with minor adverse events such as transient gastrointestinal discomfort and mild allergic reactions, without any severe complications. These women received herbal decoctions twice daily for at least 3 months and up to 24 months. Other randomized controlled trials and observation-based clinical studies have also been conducted in patients with primary or secondary dysmenorrhea and have reported few or no serious side effects associated with various herbal remedies (Li et al., 2020; Nazar and Usmanghani, 2006; Mirabi et al., 2014; Ji et al., 2020; Jang et al., 2009; Flower et al., 2009; Behmanesh et al., 2019).

Regardless of the safety outcomes reported in clinical studies, their applicability in real-world settings may vary when treatments are administered to large and diverse populations. This study is the first to evaluate the safety of HMDs for the treatment of dysmenorrhea using nationwide real-world data made available through the HMD benefit-expansion initiative and centralized health insurance claims data in Korea.

In this study, the five most frequently prescribed herbal formulas were Hyeonbuigyeong-tang (13.6%), Gagamjogyeong-tang (8.3%), Gagamojeok-san (6.8%), Danggwijakyak-san (6.2%), and Bojungikgi-tang (5.5%), which together represented more than one-third of all prescriptions from the HMD initiative. These five formulations collectively contained 37 distinct medicinal herbs. Of these, the most prevalent were *Angelicae Gigantis*/*Sinensis Radix*, *Paeoniae Radix* Alba/Rubra, Cnidii/*Chuanxiong Rhizoma*, *Cyperi Rhizoma*, and *Corydalis Tuber*/Rhizoma, in descending order of frequency.

Evidence from clinical and preclinical studies supports the safety of these commonly used herbs. Yun et al. (2015) reported no significant genotoxicity of *Angelica gigas* extract in either *in vitro* or *in vivo* rat models, suggesting a favorable safety profile for oral administration of *Angelica gigas* in traditional medicine. Shin et al. (2024) conducted a 12-week, randomized, double-blind, placebo-controlled clinical trial to evaluate the safety of *Lactiplantibacillus plantarum* SKO-001, a probiotic strain derived from *Angelica gigas*, and found no severe adverse events associated with its use.

Studies have documented the safety of formulations and extracts based on *Paeoniae Radix* Alba/Rubra (Liu et al., 2023; Mu et al., 2024), Cnidii/*Chuanxiong Rhizoma* (Man et al., 2012; Liu et al., 2025), *Cyperi Rhizoma* (Xue et al., 2023), and *Corydalis Tuber*/Rhizoma (Du et al., 2022). Our findings align with this existing evidence and further extend the current knowledge to the real-world application of herbal formulations in decoction forms in clinical settings. However, further research is warranted to understand the long-term safety and potential interactions between herbal medicines.

To strengthen causal inference, we implemented a quasi-experimental difference-in- differences (DID) design combined with coarsened exact matching (CEM). CEM created a comparison cohort of nonusers balanced on sociodemographic and clinical factors; DID then estimated pre-/post-changes attributable to initiation of the reimbursed HMD benefit, rather than baseline differences between groups. Safety outcomes were defined *a priori* from National Health Insurance claims and included healthcare utilization related to hepatic and renal toxicities, allergic reactions, emergency department visits, and hospitalizations.

These findings offer a scalable model for health policymakers in other countries. Pharmacovigilance should ideally begin at program launch rather than post-expansion. We propose a claims-enabled framework—comprising standardized coded safety endpoints, routine (e.g., monthly) signal detection, and a pre-specified escalation from statistical signals to targeted chart review. For international health systems, adopting this approach can verify safety in near real-time and trigger rapid adjustments to reimbursement criteria, prescriber standards, and clinical guidelines. This ensures that coverage policies of herbal medicine globally remain evidence-responsive while safeguarding patients in routine practice.

However, this study had several limitations. First, our analysis focused on assessing the overall safety of HMD prescriptions for dysmenorrhea as provided through a nationwide initiative rather than isolating the effects of individual herbs or formulas. Consequently, the observed safety associations apply to the overall use of HMD rather than to specific ingredients or formulations. Future research should more closely examine the safety profiles of specific herbal components and individualized prescriptions. Next, since our study relied on claims data, we lacked access to biochemical markers for hepatic and renal function, as well as immune responses, which could have provided more detailed safety assessments. In addition, our measures could not account for over-the-counter purchases of acetaminophen or ibuprofen, which may have led to underestimation of total use. Lastly, several outcome estimates had wide confidence intervals, indicative of the limited number of rare adverse events observed. Although the results did not show increased risk, this statistical uncertainty underscores the necessity for larger sample sizes or longer follow-up periods to obtain more definitive evidence. In addition, future studies should consider more detailed analyses using subgroups defined by age, comorbidity status, treatment intensity, or alternative exposure definitions to further validate the findings.

## Conclusions

In a national real-world setting, reimbursement of individually prescribed herbal medicine decoctions for dysmenorrhea was not associated with an increased risk of severe adverse events. These findings support the integration of personalized herbal medicine under National Health Insurance coverage do not raise additional safety concerns, provided that standardized reporting and claims-based pharmacovigilance remain in place. Nevertheless, the observational nature of the dataset and the limited clinical granularity inherent to claims records warrant cautious interpretation. Further studies incorporating prospective designs, predefined safety margins, and detailed clinical assessments are needed to strengthen the evidence base.

From a policy perspective, the results inform coverage criteria and guideline pathways that position herbal medicine as a safety-compatible option within dysmenorrhea care. Future work should deliver formulation-level safety profiles, dose–duration analyses, and subgroup evaluations to guide precision reimbursement and clinical decision-making.

## Data Availability

The data that support the findings of this study are available from the HIRA (Health Insurance Review & Assessment Service), but restrictions apply to the availability of these data, which were used under license for the current study and are not publicly available. Requests to access these datasets should be directed to https://opendata.hira.or.kr/home.do.
