# Supplementary material for: Safety of individualized herbal medicine for dysmenorrhea: pharmacovigilance from South Korea’s national pilot
Source: Front Pharmacol. 2026 Feb 9;17:1722249. doi: 10.3389/fphar.2026.1722249 (PMC12926767; doi:10.3389/fphar.2026.1722249)
Supplement: Supplementary file 1 [file Table1.docx]

**Supplemental Tables**

**Table S1. Robustness of analysis: testing parallel trend assumption of difference-in-difference**

|  | **One month** | | | **Three months** | | | | **Six months** | | | | **Eleven months** | | |
| --- | --- | --- | --- | --- | --- | --- | --- | --- | --- | --- | --- | --- | --- | --- |
|  | **𝜷** |  | **SE** | **𝜷** | |  | **SE** | **𝜷** |  | **SE** | | **𝜷** |  | **SE** |
| **Hepatic failure** | | | | | | | | | | | | | | |
| Once | 3.31 |  | 59.57 | | 3.46 |  | 64.29 | 3.46 |  | 64.29 | | 3.52 |  | 53.79 |
| Twice | -5.80 |  | 119.10 | | -6.50 |  | 128.60 | -6.50 |  | 128.60 | | -6.49 |  | 107.60 |
| Post-period | -5.80 |  | 89.68 | | -6.50 |  | 96.79 | -6.50 |  | 96.79 | | -6.49 |  | 80.99 |
| Once·post | -3.31 |  | 97.75 | | -3.46 |  | 105.50 | -3.46 |  | 105.50 | | -3.52 |  | 88.28 |
| Twice·post | 5.80 |  | 171.30 | | 6.50 |  | 184.80 | 6.50 |  | 184.80 | | 6.49 |  | 154.70 |
| **Renal failure** |  |  |  | |  |  |  |  |  |  | |  |  |  |
| Once | -6.63 |  | 60.98 | | -0.28 |  | 0.34 | -0.20 |  | 0.24 | | -0.17 |  | 0.19 |
| Twice | 4.36 |  | 30.50 | | 0.17 |  | 0.49 | 0.25 |  | 0.35 | | 0.40 |  | 0.27 |
| Post-period | -6.62 |  | 77.99 | | -11.74 |  | 102.10 | -12.79 |  | 126.50 | | -12.85 |  | 106.50 |
| Once·post | 5.99 |  | 118.80 | | 0.28 |  | 118.00 | 0.20 |  | 146.30 | | 0.17 |  | 123.10 |
| Twice·post | -3.19 |  | 110.40 | | -0.17 |  | 187.30 | -0.25 |  | 232.20 | | -0.40 |  | 195.40 |
| **Allergic responses** | | | | | | | | | | | | | | |
| Once | -0.27 |  | 0.19 | | 0.02 |  | 0.11 | 0.10 |  | 0.09 | | 0.01 |  | 0.07 |
| Twice | 0.11 |  | 0.28 | | -0.13 |  | 0.17 | -0.23 |  | 0.14 | | -0.08 |  | 0.11 |
| Post-period | -0.58 |  | 0.25 | | -1.12 |  | 0.19 | -0.88 |  | 0.13 | | -14.71 |  | 86.54 |
| Once·post | 0.05 |  | 0.31 | | -0.03 |  | 0.21 | -0.15 |  | 0.15 | | -0.01 |  | 100.10 |
| Twice⨯post | 0.17 |  | 0.45 | | -0.06 |  | 0.35 | 0.22 |  | 0.25 | | 0.08 |  | 158.80 |
| **Admissions to non-traditional medicine hospitals** | | | | | | | | | | | | | | |
| Once | 0.05 |  | 0.16 | | 0.08 |  | 0.08 | 0.03 |  | 0.07 | | -0.03 |  | 0.05 |
| Twice | -0.15 |  | 0.26 | | -0.17 |  | 0.14 | -0.19 |  | 0.11 | | -0.08 |  | 0.08 |
| Post-period | -0.52 |  | 0.21 | | -0.73 |  | 0.12 | -0.63 |  | 0.09 | | -15.75 |  | 107.00 |
| Once·post | -0.12 |  | 0.24 | | -0.17 |  | 0.14 | -0.11 |  | 0.11 | | 0.03 |  | 123.70 |
| Twice·post | 0.52 |  | 0.37 | | 0.14 |  | 0.23 | 0.13 |  | 0.17 | | 0.08 |  | 196.40 |
| **Emergency visits** | | | | | | | | | | | | | | |
| Once | -0.25 |  | 0.16 | | -0.11 |  | 0.09 | -0.12 |  | 0.07 | -0.08 | |  | 0.06 |
| Twice | 0.32 |  | 0.22 | | -0.10 |  | 0.15 | -0.13 |  | 0.11 | -0.13 | |  | 0.09 |
| Post-period | -1.45 |  | 0.30 | | -1.41 |  | 0.17 | -1.41 |  | 0.13 | -15.70 | |  | 117.40 |
| Once·post | 0.33 |  | 0.35 | | 0.05 |  | 0.20 | 0.17 |  | 0.15 | 0.08 | |  | 135.70 |
| Twice·post | -0.16 |  | 0.54 | | 0.25 |  | 0.30 | 0.05 |  | 0.25 | 0.13 | |  | 215.40 |

Note: ^***^ p<0.001, ^**^ p<0.01. Outcomes measured at one, three, six, and eleven months, starting from 30 days after one year before the index date, were compared to those measured at each corresponding period, starting from 30 days after two years before the index date. This analysis was conducted to assess the validity of a parallel trend assumption. The estimation of renal failure at one month was not possible as it did not occur within the designated time frame, starting from 30 days after both one year and two years before the index date.
